# Comprehensive dynamic and kinematic analysis of the rodent hindlimb during over ground walking

**DOI:** 10.1038/s41598-022-20288-3

**Published:** 2022-11-16

**Authors:** Jack Dienes, Brody Hicks, Conrad Slater, Kevin D. Janson, George J. Christ, Shawn D. Russell

**Affiliations:** 1 Laboratory of Regenerative Therapeutics, Biomedical Engineering Department, University of Virginia, MR5, Rm 1133, 415 Lane Road, Charlottesville, VA 22908 USA; 2 Motion Analysis and Motor Performance Laboratory, Mechanical and Aerospace Engineering Department, University of Virginia, 122 Engineer’s Way, 640 Kirtley Lane, Suite 103, Charlottesville, VA 22903 USA; 3 Department of Orthopaedic Surgery, University of Virginia, 2280 Ivy Rd, Charlottesville, VA 22903 USA

**Keywords:** Translational research, Biomedical engineering

## Abstract

The rat hindlimb is a frequently utilized pre-clinical model system to evaluate injuries and pathologies impacting the hindlimbs. These studies have demonstrated the translational potential of this model but have typically focused on the force generating capacity of target muscles as the primary evaluative outcome. Historically, human studies investigating extremity injuries and pathologies have utilized biomechanical analysis to better understand the impact of injury and extent of recovery. In this study, we expand that full biomechanical workup to a rat model in order to characterize the spatiotemporal parameters, ground reaction forces, 3-D joint kinematics, 3-D joint kinetics, and energetics of gait in healthy rats. We report data on each of these metrics that meets or exceeds the standards set by the current literature and are the first to report on all these metrics in a single set of animals. The methodology and findings presented in this study have significant implications for the development and clinical application of the improved regenerative therapeutics and rehabilitative therapies required for durable and complete functional recovery from extremity traumas, as well as other musculoskeletal pathologies.

## Introduction

The rat hindlimb is a frequently utilized model system for studying diverse conditions/pathologies spanning volumetric muscle loss^1–4^, nerve injury^5–8^, ligament injury^9–12^, and osteoarthritis^13–15^. These studies have been very effective in demonstrating the translational potential of regenerative therapeutics, changing our approach to pre-clinical research from evaluating only the local impact of an injury or treatment to evaluating the entire systemic response. These advances have opened the door to numerous future studies in the realm of regenerative therapeutics and rehabilitation, that are designed to better understand and predict the outcomes of musculoskeletal pathologies modeled in the rat hindlimb.

In this setting, spatiotemporal parameters^13,14,16–21^ and ground reaction forces^14,22–28^ (GRFs) utilize well characterized methodologies, are easy data to obtain, and have both been collected on rats during over-ground walking. These methods are the most sensitive to detecting changes in rat walking mechanics and weight distribution and have been shown to provide insight into compensation mechanisms related to spinal and knee injuries. Joint kinematics have also been investigated extensively, but there is a lack of congruence in the literature due to vast methodological differences. These inconsistencies include small group sizes, low volumes of analyzed gait cycles, and highly variable data collection and reconstruction protocols, each of which can contribute to the range of outcomes observed in the kinematic literature. But despite the broad differences in the literature, kinematics have been used on a case-by-case basis to evaluate changes in joint motion due to arthritis, spinal injury, or varied walking conditions.

As such, kinematics are an incredibly useful evaluative tool. However, muscle moments (kinetics) are the driving force behind movement patterns, and to date kinetics have not been reliably calculated in rodent models and researchers have primarily relied on spatiotemporal parameters^13,14,16–21^, ground reaction forces (GRFs)^14,22–28^, or 1-D kinematics (joint angles)^17,29–33^ to quantify gait changes. In this regard, 3-D kinetics have long been the gold standard of human motion capture and movement analysis, and if these methods are effectively applied to rats then pathological effects and treatment efficacy can be more extensively evaluated. Kinetics offer insight into neuromuscular recruitment strategies and joint loading that no other analysis can provide. Kinetics are the gold standard for human motion analysis for that reason, they allow you to see how the muscles and joints in a system are working to actuate a motion and quantify the forces being experienced at each of the joints. They also provide insight into joint loading, compensation patterns, joint power, and efficiency of motion that would otherwise be undetectable.

The ability to calculate this data for pre-clinical rat models of any neuromusculoskeletal pathology will be beneficial. However, this work is specifically relevant to our continuing efforts to expand the application of multiscale biomechanical metrics for improved evaluation of volumetric muscle loss (VML) injury and repair. Here improved maximum isometric torque has been the primary evaluative metric, even though increases in this measure alone do not necessarily result in improved functional outcomes^34–38^.With the large volume of pre-clinical studies being performed on the rat hindlimb to assess muscle, nerve, tendon, and joint injuries, kinetic insight into the extent of injury and the road to recovery would be instrumental in fine-tuning rehabilitative and regenerative therapies.

The primary objective of this study was to develop the necessary modeling methods, as well as a robust database for thorough analysis of the rat hindlimb during normal over-ground walking. To this end, we added all the previously described metrics to our own developed method for calculating 3-D joint-by-joint kinetics. Because of the overall utility of spatiotemporal parameters and GRFs, we have made a point to report them within our normative database. In short, in this report, we implemented these advanced motion capture and modeling techniques to capture concurrent marker and GRF data for rat locomotion. We were then able to calculate 3D joint kinetic data, which represents a breakthrough in rodent gait analysis. Our current findings confirm and extend previous work, and have major implications for improved treatment of extremity trauma. Specifically, we posit that the information obtained from this more comprehensive biomechanical approach, including thorough kinetic analysis, has the potential to maximize functional recovery and minimize the adoption of compensatory gait patterns by providing detailed insight into the true mechanisms responsible for diminished function following various injuries and pathologies.

## Methods

### Experimental outline

A total of 24 female Lewis rats (Charles River Laboratories) weighing 180.0 ± 7.8 g at 12 weeks of age were tasked with walking on a 2.7 m instrumented walkway. Motion data was collected and analyzed using a combination of Vicon Nexus motion capture software and OpenSim musculoskeletal modeling. Concurrent GRF data was acquired from these trials using ATI Nano43 sensors with a load range ± 9 N (ATI Industrial Automation NC), load cells were mounted with top plates (85 × 50 mm) flush to the walkway surface. Trials were excluded if the rats stopped in the middle of the collection volume, turned around, accelerated below or beyond a lateral sequence walk (17 cm/s < walking velocity < 48 cm/s^39^). Spatiotemporal parameters were acquired from marker positions calculated by Vicon Nexus. Kinetics were calculated by performing inverse dynamics to combine the joint angle and GRF data in the OpenSim model^40,41^. Spatiotemporal parameters, joint kinematics, GRFs, joint moments, and joint powers were compiled and averaged to create a normative database for rodent gait. Data presented represents a mean of 8.6 ± 3.6 steps/rat averaged for kinematic and spatiotemporal calculations and a mean of 2.1 ± 1.1 averaged foot strikes recorded on the force plates per rat for kinetic calculations.

### Animal care

This study was conducted in compliance with the Animal Welfare Act, the Implementing Animal Welfare Regulations, in accordance with the principles of the Guide for the Care and Use of Laboratory Animals, and in accordance with ARRIVE guidelines. The University of Virginia Animal Care and Use Committee approved all animal procedures. Animals were pair housed in a vivarium accredited by the American Association for the Accreditation of Laboratory Animal Care, and they were provided with food and water ad libitum.

### Acquisition of motion data and ground reaction forces

**Figure 1:**
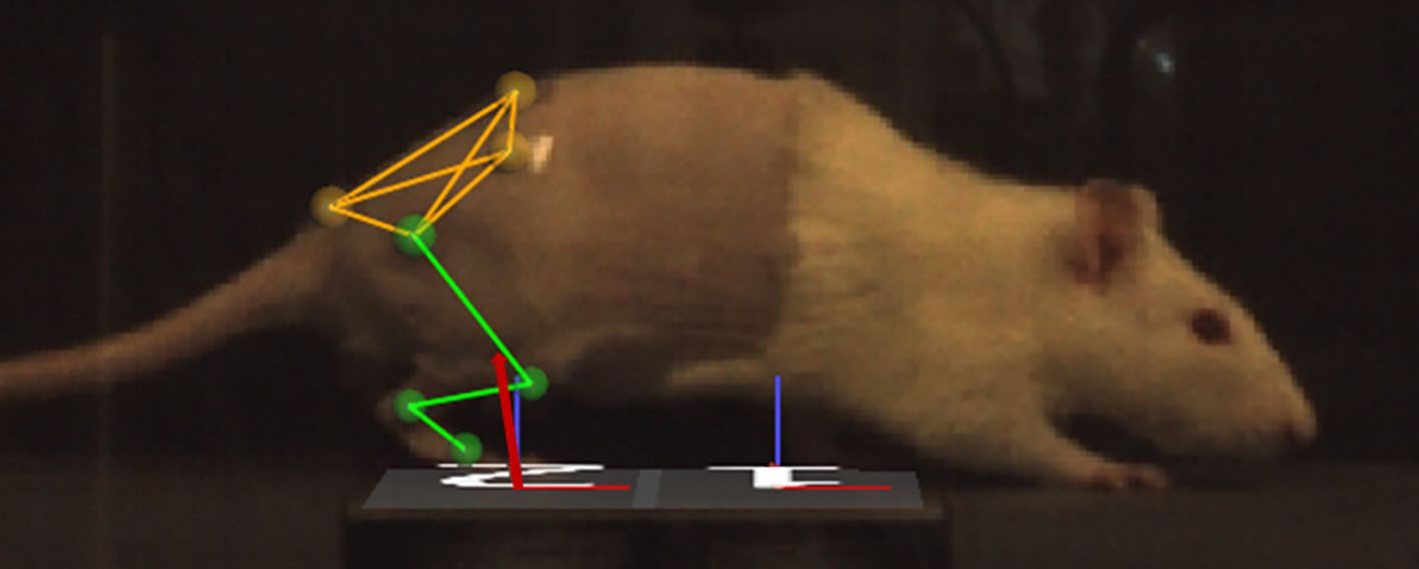
Vicon Nexus 2.7.1 3-D overlay of motion capture marker placements. 3 mm markers were applied to the lateral knee, ankle, and fifth metatarsal (TOE) of the female Lewis rats. 5 mm markers were applied to the spine, hip, right anterior superior iliac crest (RASI), and the tail. Motion capture data was collected at 200 Hz and ground reaction force (GRF) data was collected at 1000 Hz. Data was reconstructed using Vicon Nexus 2.7.1 resulting in overlays of joint positions and GRF vector projections as shown above.

Rats were briefly anesthetized with 2–3% isoflurane in 100% oxygen prior to motion capture and shaved to allow proper placement of the motion capture marker set^40^ illustrated in Fig. 1. 3 mm and 5 mm reflective markers were placed on the bony landmarks of the left anterior superior iliac crest (LASI), right anterior superior iliac crest (RASI), spine (L6 vertebra), tail (5th caudal vertebra), hip, lateral knee, ankle, and distal end of the fifth metatarsal. Rats were allowed to recover from anesthesia on a heated mat before being placed in the instrumented walkway. Marker data was collected using a 7-camera setup (Vicon, Oxford Metrics, Oxfordshire, ENG) collecting at 200 Hz and GRF data was collected at 1000 Hz. A threshold of 0.07 N, normal to the surface, was used to define foot contact and toe off for each step. After data collection the animals were returned to the vivarium.

### Limb morphometrics and MoI/CoM calculations

We have previously developed a kinematic model for the rat hindlimb^40^, but in order to calculate accurate kinetic data, it is necessary to have both GRF data and inertial data of the rat limbs. To determine inertial parameters, rat limb morphometrics were compiled from the fresh cadavers, necessitating the use of an independent group of 17 female Lewis rats of the same strain, lot, and provider. For each segment of the hindlimb (thigh, shank, foot), lengths (joint center to joint center), and masses were measured, normalized by body weight, and averaged (n = 34 limbs) to obtain morphometric data. We then scaled the normalized data geometrically to extrapolate values for animals of different body weights. This data was used to inform accurate weights for each limb segment within the OpenSim model prior to scaling of the model to each individual rat. Centers of mass (CoM) and moments of inertia (MoI) were determined by performing laser surface scans of the left and right hindlimbs of a representative sample of 5 female Lewis rats. The scans were meshed, smoothed, and reconstructed using Meshmixer software (Autodesk, USA). Reconstructed limbs were then converted to solids and segmented into thigh, shank, and foot sections using Fusion 360 software. Segment masses were extrapolated based on the body weight of the scanned animal and equations determined from the rat limb morphometric database. Once these parameters were established, CoM and 3-D MoI data for each limb segment was calculated by Fusion 360 (Autodesk, USA). CoM and MoI values were normalized to body weight and limb length then averaged so they could be applied to animals over a range of sizes and ages. These inertial measurements were added to the OpenSim model prior to performing inverse kinematics and inverse dynamics.

### Inverse kinematics

Gait events and marker identification was completed in Nexus (Vicon, Oxford Metrics, Oxfordshire, ENG) and marker position data was lowpass filtered at 15 Hz (fourth order, two-way Butterworth). Inverse kinematic modeling was performed in OpenSim using a validated rat hindlimb kinematic model^40,41^. Calculated CoM and MoI data for the limb segments of each rat was programmed into the kinematic model prior to modeling any walkway trials. This model consisted of four segments (pelvis, femur, tibia, foot) and each joint was modeled as free, ball, revolute, and revolute, respectively. This facilitated full 3D analysis of hip and pelvis kinematics and kinetics while reducing the number of motion markers required, the knee and ankle joint were limited to flexion and extension due to their small size and marker placement.

### Inverse dynamics

Kinetics were calculated using inverse dynamics by pairing 3D GRF data with concurrently captured motion data using a validated rat hindlimb kinematic model (v.0.2) in OpenSim^41^. For all simulations the segment coordinates and their relationship to bony landmarks were defined in the method of Johnson^40^, in addition all joint angles were calculated using a Sagittal, Frontal, Transverse Euler sequence. Calculated CoM and MoI data for the limb segments of each rat was programmed into the kinematic model prior to modeling any walkway trials. Marker data and measured GRFs were extracted from Nexus for each trial. Marker data was lowpass filtered at 15 Hz and GRFs were lowpass filtered at 100 Hz (fourth order, two-way Butterworth). This data was then imported into OpenSim and models were run using the inverse dynamics solver, using the methods of Winters^42^, resulting in 3D joint moments at the hip and sagittal plane joint moments at the knee and ankle. Kinetics for each rat, at each timepoint, were normalized by body mass. Utilizing the joint moments and the sagittal plane joint kinematic data, power absorption/dissipation at each joint was calculated in the sagittal plane over the whole stride using Eq. (1).

1 $$P \, = \, M\omega$$

where *P* is joint power, *M* is joint moment, and *ω* is joint angular velocity.

## Results

We successfully collected morphometric, spatiotemporal, kinematic, and 3D ground reaction force data on twenty-four (12)-week old female Lewis rats. The joint angles, joint moments, and power data are reported over a full gait cycle, heel strike to heel strike of the right leg.

### Spatiotemporal parameters and morphometrics

Morphometric data for segment lengths as well as spatiotemporal parameters for stride length, velocity, cadence, and stance percentage are shown in Table 1.

**Table 1:** Spatiotemporal parameters and morphometric measurements.

Parameter	Value
Stride Length (mm)	122.4 ± 4.8
Velocity (cm/sec)	30.3 ± 3.4
Cadence (steps/min)	294.2 ± 30.7
Stance Percentage (%)	63.9 ± 3.6
Thigh Segment Length (mm)	31.5 ± 2.7
Shank Segment Length (mm)	32.3 ± 2.2
Foot Segment Length (mm)	35.9 ± 2.2

### Kinematics

**Figure 2:**
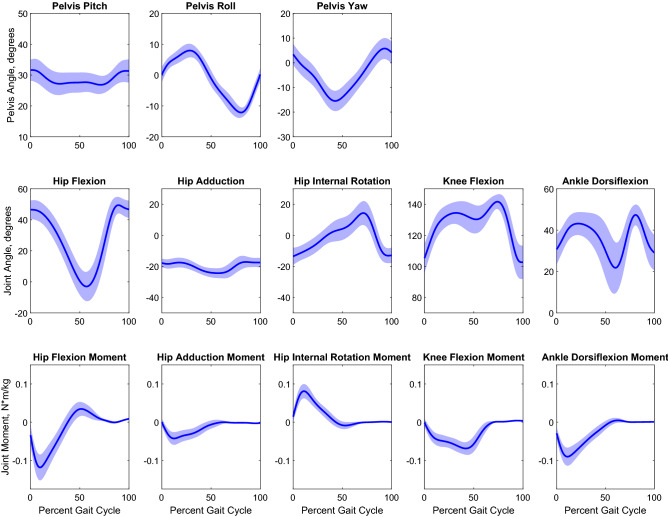
Kinematic (joint angles, top 2 rows) and kinetic (joint moments, bottom row) curves for healthy female Lewis rats. Curves are shown as mean + /− 1STD for pelvic rotation, hip flexion angle/moment, hip adduction angle/moment, hip internal rotation angle/moment, knee flexion angle/moment, and ankle dorsiflexion angle/moment.

Kinematic trajectories of each joint are reported in Fig. 2. The mean pelvis excursions were 6.90 ± 2.46 deg pitch, 20.86 ± 3.57 deg roll and 21.90 ± 3.25 deg yaw. The ranges of motion for hip flexion, adduction, and rotation were 54.0 ± 7.7, 10.1 ± 3.8, and 30.1 ± 5.9 degrees. The average maximum flexion of the hip was 50.1 ± 5.3 degrees. The ranges of motion for knee and ankle flexion were 40.6 ± 8.2 and 30.0 ± 9.9 degrees. Average maximum flexions for the knee and ankle were 142.7 ± 4.4 and 48.6 ± 3.8 degrees.

### Ground reaction forces

**Figure 3:**
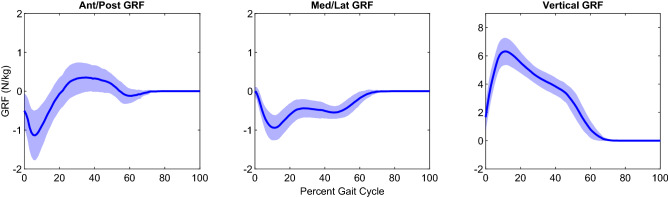
Ground reaction force (GRF) curves for healthy female Lewis rats. Curves are shown as mean + /− 1STD for all three axes (x, y, z). The X-axis is represented by the anterior/posterior forces, the Y-axis is represented by the medial/lateral forces, and the Z-axis is represented by the vertical forces. Data was compiled from load-cell footstrikes from 24 female Lewis rats.

GRFs measured from the load cells are shown in Fig. 3. The average peak anterior and posterior forces were -1.298 ± 0.697 N/kg and 0.499 ± 0.384 N/kg. The average peak medial and lateral forces were 0.047 ± 0.077 N/kg and -1.092 ± 0.319 N/kg. The average peak vertical force was 6.628 ± 0.758 N/kg.

### Joint moments and power

**Figure 4:**
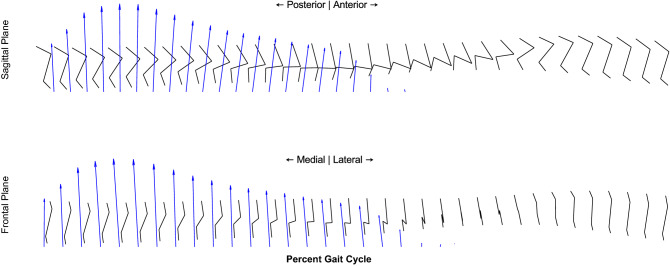
Stick figure of sagittal (top) and frontal (bottom) joint configurations and ground reaction force. One typical stride shown, initial heel strike (left) to final heel strike (right).

**Figure 5:**
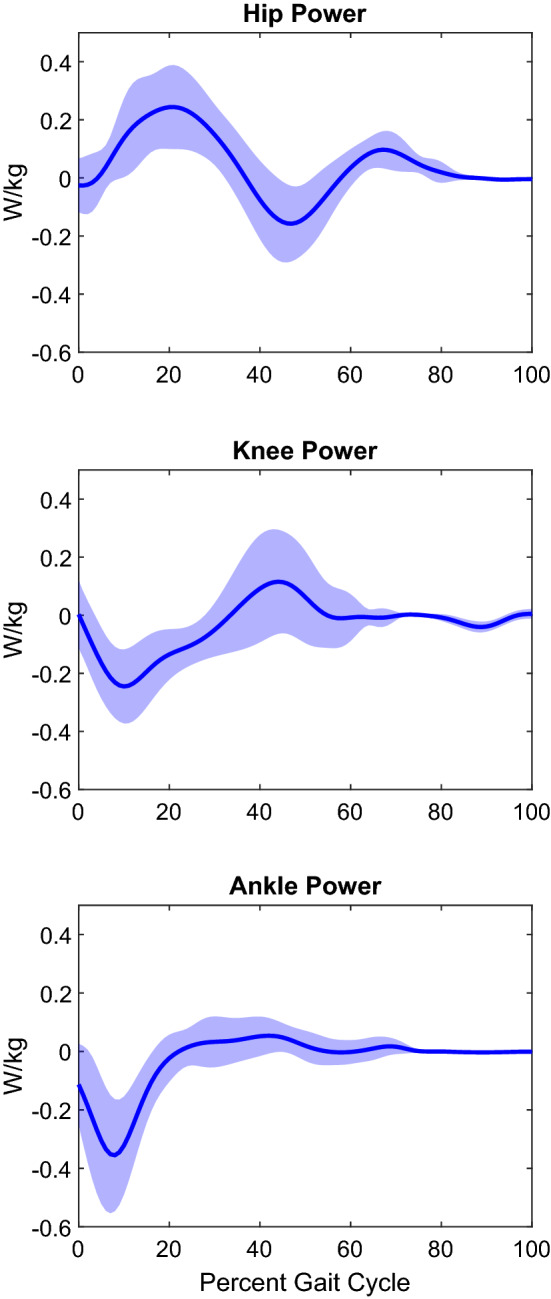
Joint power curves for healthy female Lewis rats. Curves are shown as mean + /− 1STDfor all three joints (hip, knee, ankle) for 24 healthy female Lewis rats. All power plots represent the sagittal plane (i.e. flexion/extension) in W/kg.

Joint moments calculated from concurrently recorded joint kinematics and GRFs are shown in Fig. 2, Fig. 4 shows a stick diagram for a typical gait cycle. The average peak flexion and extension moments about the hip were 0.040 ± 0.016 and -0.122 ± 0.035 Nm/kg. The average peak adduction and abduction moments about the hip were 0.005 ± 0.006 and − 0.045 ± 0.016 Nm/kg. The average peak internal rotation moment about the hip was 0.084 ± 0.019 Nm/kg. The average peak extension moment about the knee was − 0.072 ± 0.016 Nm/kg. The average peak flexion and extension moments about the ankle were 0.007 ± 0.005 and − 0.093 ± 0.023 Nm/kg. Power, in the sagittal plane, was calculated from joint moments and angular velocity data extracted from kinematics, and the results are shown in Fig. 5. Average peak generation and absorption power for the hip was 0.326 ± 0.147 W/kg and − 0.226 ± 0.126 W/kg. Average peak generation and absorption power for the knee was 0.200 ± 0.187 W/kg and − 0.316 ± 0.128 W/kg. Average peak generation and absorption power for the ankle was 0.100 ± 0.061 W/kg and − 0.374 ± 0.221 W/kg.

## Discussion

The methods and data developed in this work should expand the scope for evaluation of pre-clinical rat studies to include any neuromusculoskeletal pathology of interest modeled in the rat hindlimb. Moreover, this analysis can now be accomplished at a much deeper level of biomechanical and physiological detail. As far as we are aware, this is the first study to report 3D kinematics and 3D kinetics from concurrently recorded motion capture marker and GRF data. This work also compiled data from a larger cohort of animals than previous kinematic or kinetic studies (24 vs. an average of 11^17,19,21,29–33,40,43–51^), thus generating a more comprehensive dataset. The current study also extends previous work that has documented measurable kinematic differences after surgical creation of VML injuries^40^. More specifically, the methods developed herein will allow us to expand our biomechanical analysis to the 3-D kinetic implications of these injuries; including but not limited to, changes in motor control, neuromuscular recruitment patterns, and compensation strategies in response to VML injury or other musculoskeletal pathologies. As such, we can now analyze the impact of injury and treatment on both a joint-by-joint and systemic basis, providing a complete picture of the response to injury, as well as the recovery timeline. Furthermore, these new kinetic measures will allow us to connect functional recovery at the whole animal level (motion) to observations at the cell and tissue level (both histology and muscle contractility)—providing unprecedented multiscale biomechanical insight into the mechanisms responsible for both VML-related functional deficits, as well as mechanisms of functional recovery upon application of regenerative therapeutics.

## Spatiotemporal parameters

Though kinematics and kinetics were the primary outcomes of this study, spatiotemporal parameters remain important metrics. Spatiotemporal data includes parameters such as stride length, velocity, cadence, and time in swing, stance, and double vs. single support, all of which have been successfully calculated for both treadmill and over-ground walking^13,14,16–21,25,31,52–58^. This data is important to evaluate because it is the most sensitive to changes in gait mechanics, despite providing little insight to the mechanisms driving the change. From the list above, walking speed and step length are the best identifiers of gait abnormalities, but they offer little insight to underlying cause. Our recorded values (shown in Table 1) are similar to previously reported values for rat walking, demonstrating that the rats in this study were moving at a reasonable self-selected pace. Specifically, the average walking speed of 30.3 ± 3.4 cm/s falls well within the range of previously published literature values for healthy walking of 22–71 cm/s^16,17,19,21,25,56–58^. In addition, the mean stance percentage of 63.9 ± 3.6% compares favorably to a range of 60–73.9%^17,18,55,58^, as well as mean stride length of 122.4 ± 4.8 mm, when compared to a reported range of 82.5–150mm^16,17,20,21,54,55,58^.

## Ground reaction forces (GRFs)

GRFs (Fig. 3) provide insight on the amount of body support, the impacts of injury, and balance during ground contact in the stance phase of the gait cycle. They also identify where and when the rats absorb and generate propulsive force during heel strike and toe-off, as well as how stable the rats are during the contralateral swing phase as indicated by the lateral forces. GRFs for normal locomotion have been obtained for healthy rats^14,22–28^, but previous studies collecting GRF data have varied in the velocity of their control animals (30–85 cm/s^14,23,28^) indicating that some of these animals were likely not walking. The data presented here was collected on animals with an average moving velocity of 30.3 cm/s, within the threshold for a lateral sequence walk (< 48 cm/s^39^), thereby providing a basis for GRFs during normal walking. On average, the vertical GRFs in this study represent 67.5% of body weight and falls in the range of previously reported vertical GRF values (65–87% of BW^22–28^). These vertical forces are highest in early stance and then steadily decrease, which is different than what is typically seen in humans. The anterior–posterior GRFs represent braking/acceleration forces and pass just above and below the zero-level (braking 13.2% vs 4–15% of BW^22–24,27,28^, acceleration 5.1% vs 2–16% of BW^22–24,27,28^). Similar to humans, these forces show that the rats decelerate in early stance and accelerate in late stance. The medial–lateral GRFs pass below the zero-level as the rat lands on the outside of their foot at heel strike and rolls off the second phalange at toe-off (medial 0.47% vs 0–3%, lateral 11.2% vs 5–8%^22–24,28^). The fact that the medial–lateral GRF is always pushing back towards center is likely exaggerated by the wide step width of rats relative to what is typically seen in human subjects. The high number of foot strikes obtained in this study, low variance, and similarity to published data show the findings are representative of normal over ground walking at self-selected pace for healthy rats. However, as noted earlier, GRFs are a whole limb measurement. It is only when combined with kinematics that GRFs provide truly informative data through the calculation of joint-by-joint moments.

## Kinematics

Joint kinematics have been examined extensively in rat models^17,29–33,44^ to evaluate changes in joint motion due to muscle, nerve, or joint injuries. 3-D kinematics, as shown by João^44^, provide significant data for gait evaluation because they easily characterize classic compensation patterns (such as circumduction or vaulting) while also having enough precision to identify smaller changes in the motion of the hip, knee, or ankle that could have longer-term impacts such as osteoarthritis. Kinematics also provide the foundation for kinetic and energetic analysis by providing information on the angular acceleration and angular velocity of the limb segments. But as previously mentioned, there is a lack of congruence in the kinematic literature due to vast methodological differences. Previously reported values for sagittal plane kinematics vary significantly in their raw angles, primarily due to limitations in motion capture techniques. For example, some groups used permanent ink dots rather than reflective markers to track joint locations and/or only utilized 2-D motion capture to evaluate solely the sagittal plane.

Further, there are frequent differences in model definitions in the kinematic literature, such as neutral joint angle definitions. Some groups define the neutral angle as 0° and others define neutral as 90°, leading to a disconnect in how data is reported. To address the latter point, we defined our neutral position consistent with existing protocols for human movement analysis^59^. Similarly, another limitation of kinematic analysis in rodents has been the inaccuracy of modeling the motion of the knee due to the extreme amount of skin artifact. Bauman et al. ^29^ used 2-D X-ray fluoroscopy to show that there are broad differences in the kinematics of the knee when using skin-derived, triangulated, and bone-derived angle measurements. The data presented here compares favorably to the shape and motion of bone-derived knee kinematics reported by Bauman^29^ relative to their own skin-derived kinematics. We only observed two differences when comparing the two datasets. The first difference was a slight offset in our hip flexion angle due to the aforementioned differences in neutral angle definition (we used the sacroiliac crest and they used the caudal ischium). Second, there is a discrepancy in the knee angle at mid-stance that could be attributed to the difference in collecting 3-D versus 2-D motion data. We observed significant out of plane movement of the knee joint center which would not be accounted for when using single plane fluoroscopy (Fig. 2). A limitation of this study is the omission of the toe segment. However, even though rats use a digitigrade gait, most other studies also exclude this segment in rat gait. The rationale for so doing is likely related to the small scale and independent motion of the toes—decreasing the feasibility of accurately tracking the segments. Current work is focused on including some motion of the toes.

## Kinetics

As mentioned, joint kinetics are the gold standard for the evaluation of human biomechanics. They provide information on the internal forces being experienced on a joint-by-joint basis that cannot be captured with any other evaluative method. This information is significant because the same angular joint motion can be produced using drastically different muscle activation patterns, and many times those alternative patterns are the driving cause of long-term comorbid joint conditions. Kinetics also reveal the working relationship between joints, as a deviation at one joint in a system nearly always results in a deviation at another joint, many times hidden in the contralateral limb. Further, the ability to do kinetic analysis of gait informs everything from the etiology of a disease to treatment decisions, treatment outcomes, and the ultimate health of the entire system. Recovery from a musculoskeletal or neural injury is more than just the local recovery from the immediate presenting issue, it is a systemic undertaking that requires a comprehensive evaluation to assess the true extent of recovery.

Three studies have previously attempted to characterize rat gait kinetics, and they were conducted by Bennett et al. ^31^ and Andrada et al. ^25,26^. These studies were performed on 5 animals and 2 animals, respectively, and calculated joint moments for the hip, knee, and ankle in only the sagittal plane. In addition, Wehner^60^ developed 3D joint moments, but because they did not measure GRFs they used measured kinematics combined with published GRF’s to calculate joint loading. The curve shapes of the kinetic analysis presented here align well with the pre-surgery moments presented by Bennett^31^ and the shapes of the kinetic curves of the hip and ankle compare well to both sets of data presented by Andrada^25,26^ with our peak values falling within the range of their reported values. There is some disparity in the shape of the knee torque graphs, but because Andrada^25,26^ did not publish their kinematic curves it is also difficult to pinpoint the exact source of these differences. However, they did note that their imaging in 2D would impact their sagittal plane angles. By design, imperfect knee angles would lead to incorrect knee moments. In our current study, the reported methods and data permitted calculation of joint moments in all three planes at the hip (flexion/extension, ab/adduction, internal/external rotation). Because of the combination of consistent 3D kinematic curves and low variance GRFs in this study, the calculated kinetic results in all three planes are reliable and provide a solid benchmark for 3D rat gait analysis moving forward.

Joint power analysis is an under-researched area with respect to rodent gait studies, but the relative shapes of our power plots for the hip, knee, and ankle (Fig. 5) compare favorably to the three other published datasets in Bennett et al. ^31^ and Andrada et al. ^25,26^. However, the values reported by Bennett^31^ for normalized power are extremely high (peaks greater than 60 W/kg). This may be due to a failure to convert from degrees/second to radians/second for angular momentum values, because both their kinematic and kinetic curves present reasonable data. For comparison, Bennett^31^ reports a peak hip power of ~ 60 W/kg, but this value is significantly higher than peak values reported for humans (~ 1.8 W/kg^61^), horses (~ 6 W/kg^62^), or rats in the Andrada^25,26^ studies (0.08–1.2 W/kg^25,26^). There is a clear discrepancy between Bennett’s reported values and the field, but the values presented by Andrada^25,26^ fall far more within the reasonable expected range for peak joint power of walking rats. Based on that data, our power curves fall within the range presented by Andrada^25,26^ and are on the proper scale of the expected hip, knee, and ankle joint powers during healthy, normal walking for Lewis rats.

Because the rat hindlimb is utilized as a model system for the treatment and evaluation of so many pathologies, this method of comprehensive gait evaluation should be of broad utility to the field. As far as we are aware, the data presented in this paper is the first to characterize kinetics in all three planes, providing comprehensive insight into the biomechanics of rat walking on a joint-by-joint basis. In addition, kinematic analysis in this study was conducted on a larger cohort (24 vs. an average of 11^17,19,21,29–33,40,43–51^) and included more kinetic data on both an animal basis and foot-strike basis than previous studies in the field. As such, this work extends the analytic methods available to investigators to study the rat hindlimb beyond more widely used measurements, such muscle force production, to include insight into internal forces and movement compensation patterns. Furthermore, similarities of rat gait to the human crouch gait benchmark should provide additional translational value to these investigations for improved understanding, evaluation, and treatment in humans.

## Outlook/conclusion

We are particularly excited about the potential applications of this approach to provide improved understanding of the biomechanical mechanisms responsible for functional deficits caused by extremity trauma (e.g., VML injuries), and thus, to shed new insight on potential therapeutic solutions for improved treatment of limb trauma. High impact trauma such as VML and/or peripheral nerve injury to the extremities impacts thousands of wounded warriors and civilians each year. Frequently, especially in complex compartments with multiple muscles and innervation patterns, the biomechanical impact of these injuries and the route to recovery can be difficult to precisely identify. The addition of 3D kinetic analysis provides an important new tool to gain greater insight into the “black box” of potential mechanisms responsible for the functional deficits observed in these complex injuries, as well as any compensatory neuromuscular responses/adaptations. In theory, implementing this new evaluative tool into the armamentarium of methods available should accelerate development of more effective treatments for functional restoration of extremity trauma, and ultimately significantly increase the quality of life for impacted individuals.

In this regard, we have developed a comprehensive method to analyze the full 3D kinetics and 3D kinematics of the rat hindlimb during over ground walking to provide a clearer picture of the biomechanics required for normal movement function. Our unique ability to measure gait parameters, as demonstrated in this study, facilitates a more thorough understanding of normal and healthy rodent gait. With this methodology and developed kinetic database in hand, it is possible to extend these protocols to quantify relevant functional deficits in rat models, as well as the functional effectiveness of therapeutic interventions on movement quality. Further, the normative database presented herein provides valuable data for comparison for any study making use of rat models for full biomechanical analysis (spatiotemporal parameters, GRFs, kinematics, kinetics, and energetics) of pathologies modeled in the hindlimb.

## Data Availability

The datasets generated during and/or analyzed during the current study are available from the corresponding author on reasonable request.
